# Acute Cardiotoxicity With Concurrent Trastuzumab and Hypofractionated Radiation Therapy in Breast Cancer Patients

**DOI:** 10.3389/fonc.2019.00970

**Published:** 2019-10-01

**Authors:** Mutlay Sayan, Zeinab Abou Yehia, Apar Gupta, Deborah Toppmeyer, Nisha Ohri, Bruce G. Haffty

**Affiliations:** ^1^Department of Radiation Oncology, Rutgers Cancer Institute of New Jersey, Rutgers University, New Brunswick, NJ, United States; ^2^Department of Medicine, Rutgers Cancer Institute of New Jersey, Rutgers University, New Brunswick, NJ, United States

**Keywords:** breast cancer, radiation therapy, hypofractionated breast irradiation, trastuzumab, cardiac toxicity

## Abstract

**Purpose:** Radiotherapy for patients with non-metastatic human epidermal growth factor receptor 2 (HER2) positive breast cancer is commonly administered concurrently with adjuvant trastuzumab. However, there is limited data on the use of concurrent trastuzumab and hypofractionated radiotherapy (Hypo-RT), which is now standard of care for the majority of women receiving whole breast irradiation. In this study, we compared acute cardiotoxicity rates in HER2-positive breast cancer patients treated with concurrent trastuzumab and Hypo-RT or conventionally fractionated radiotherapy (Conv-RT).

**Methods:** We performed a review of our institutional database to identify HER2-positive breast cancer patients treated with trastuzumab and Hypo-RT or Conv-RT from 2005 to 2018 who underwent serial cardiac Left Ventricular Ejection Fraction (LVEF) evaluation. Decrease in LVEF was assessed by either echocardiography (ECHO) or multiple gated acquisition (MUGA) scan performed at baseline and every 3 months during trastuzumab therapy. Significant LVEF decline was defined as an absolute decrease in LVEF of ≥10% below the lower limit of normal or ≥16% from baseline value.

**Results:** We identified 41 patients treated with Hypo-RT and 100 patients treated with Conv-RT. Median follow-up was 32 months (range, 13–90 months). Baseline median LVEF was 62% (range, 50–81%) in Hypo-RT group and 64% (range, 51–76%) in Conv-RT group (*p* = 0.893). Final median LVEF was 60% (range, 50–75%) in both groups. Three patients (7%) in Hypo-RT and five (5%) in Conv-RT group developed significant asymptomatic LVEF decline (*p* = 0.203). There was no significant difference in mean heart dose in patients who developed significant asymptomatic LVEF decline vs. those who did not in Hypo-RT (*p* = 0.427) and Conv-RT (*p* = 0.354) groups. No symptomatic congestive heart failure was reported in either group.

**Conclusions:** The rate of asymptomatic LVEF decline in patients receiving concurrent trastuzumab and Hypo-RT was low (7%) and was similar to the rate observed in patients receiving Conv-RT. Longer follow-up is warranted to assess late cardiotoxicity.

## Introduction

The human epidermal growth factor receptor 2 gene (HER2) is overexpressed and/or amplified in approximately 20% of primary breast carcinomas and was historically considered a marker of poor prognosis (1, 2). Trastuzumab is a humanized monoclonal antibody that targets the extracellular domain of the HER2 oncoprotein (3). Multiple large randomized trials demonstrated reduced recurrence rates and improved survival with the use of adjuvant trastuzumab in HER2 positive breast cancer (4–8). In 2006, trastuzumab was approved by the US Food and Drug Administration (FDA) for the adjuvant treatment of localized HER2 positive breast cancer and is now standard of care.

Trastuzumab is generally well-tolerated; however, cardiac dysfunction (congestive heart failure [CHF] or decrease in left ventricular ejection fraction [LVEF]) remains a concern. While the overall incidence of cardiac toxicity is variable, likely due to inconsistent definitions used in trials, asymptomatic LVEF decline is the most frequent form of cardiotoxicity. In most trials, significant asymptomatic LVEF decline was defined as an absolute decrease in LVEF of ≥10% below the lower limit of normal or ≥16% from baseline, with incidence rates ranging from 3.5 to 18.6% (5–7, 9). Current guidelines therefore recommend assessment of LVEF at baseline and every 3 months during the therapy (10).

Adjuvant radiation therapy (RT) is standard of care after breast-conserving surgery and in patients at high risk for local relapse who undergo mastectomy. The risk of radiation-related long-term cardiotoxicity has been shown to increase in a dose-dependent fashion (11), and as a result radiotherapy techniques have been modernized to limit cardiac exposure. Conventionally fractionated radiotherapy (Conv-RT), or delivery of 1.8–2.0 Gy daily per fraction over 5–7 weeks was previously considered standard of care for all breast cancer patients requiring adjuvant radiotherapy. More recently, updated guidelines have endorsed a hypofractionated approach (Hypo-RT) for whole breast irradiation with delivery of over 2.0 Gy daily per fraction over 3–4 weeks (12).

Despite the common concurrent use of trastuzumab and radiotherapy in breast cancer, there is limited data on the safety of concurrent adjuvant trastuzumab administration and breast radiotherapy, particularly with hypofractionated radiation therapy. Preclinical data suggests a radiosensitizing effect of trastuzumab on breast cancer cells, but whether it causes radiosensitization of normal cells is unknown (13, 14). The potential synergistic effect of concurrent trastuzumab and RT on the heart remains a major concern, and can influence the routine use of hypofractionated radiation in patients with breast cancer, particularly in patients with left sided breast cancer (15, 16). While limited series have assessed acute cardiotoxicity with trastuzumab and Conv-RT, it is unknown whether the use of Hypo-RT with trastuzumab results in the same risks (17). In this study, we evaluate and compare cardiotoxicity rates in a cohort of HER2-positive breast cancer patients treated with adjuvant trastuzumab and concurrent Hypo-RT or Conv-RT.

## Methods

### Patient Population

Using an IRB-approved protocol, we conducted a retrospective review of our institutional database to identify patients with stage I–III breast cancer from 2005 to 2018. The criteria for inclusion included: (1) HER2-positive disease; (2) receipt of Hypo-RT or Conv-RT; (3) concurrent receipt of trastuzumab; and (4) LVEF assessment by either echocardiography (ECHO) or multiple gated acquisition (MUGA) scan at baseline and every 3 months from the start of trastuzumab therapy.

### Baseline Characteristics

Baseline clinical characteristics were collected and included patient age, race, and cardiac risk factors such as BMI ≥30, age ≥55 years, history of hypertension, hyperlipidemia, diabetes, CAD, smoking, and family history. Disease-related characteristics including histology, hormone receptor status, pre-chemotherapy clinical stage, and pathologic stage were also recorded. Treatment related factors included type of breast surgery (breast conserving surgery vs. mastectomy with or without reconstruction), receipt of chemotherapy (neoadjuvant vs. adjuvant chemotherapy), hormonal therapy, and adjuvant radiation therapy (Hypo-RT vs. Conv-RT).

### Treatment

Neoadjuvant and adjuvant systemic chemotherapy and/or adjuvant hormone therapy was administered as clinically indicated in accordance with standard practices during this time interval. Breast-conserving surgery or mastectomy with or without reconstruction was performed. Axillary evaluation included sentinel node biopsy (SLNB) and/or axillary lymph node dissection (ALND).

Adjuvant RT was delivered using 3-dimensional conformal radiotherapy (3D-CRT) to the breast/chest wall with or without regional nodal irradiation. Boost was delivered as per physician discretion. Computed tomography–based treatment planning with tangential fields was used for all patients. The heart was excluded from the primary beam using multi-leaf collimator blocking. The deep inspiratory breath hold (DIBH) technique was utilized to limit cardiac exposure when clinically indicated.

Concurrent trastuzumab was administered every 3 weeks for up to 12 months at a standard dose of 2–6 mg/kg. Treatment was held if the LVEF dropped below 50% or when a patient developed any symptoms of heart failure. After a repeat evaluation at 4 weeks, if the LVEF was not recovered or in the case of confirmed congestive heart failure, trastuzumab was discontinued.

### Follow-Up and Evaluation of Toxicity

Patients were seen weekly while undergoing radiation therapy and again around 1 month after completion of radiotherapy. Subsequent radiation oncology follow ups were typically around 6 months, 1 year, and yearly thereafter. Patients typically followed up with the medical oncologists every 1–3 months while undergoing chemotherapy, every 3–6 months for the following 2 years, and every 6–12 months thereafter. Follow-up was calculated from the time of radiotherapy completion.

Toxicities were scored using the Radiation Therapy Oncology Group/European Organization for Research and Treatment of Cancer (RTOG/EORTC) scale. Cardiac toxicity was evaluated by clinical assessment of cardiac function and by LVEF on ECHO or MUGA scans at baseline before staring trastuzumab, every 3 months during trastuzumab treatment, and at 3 months post-treatment if needed. Significant asymptomatic LVEF decline was defined as an absolute decrease in LVEF of ≥10% to below the lower limit of normal or ≥16% from baseline value.

### Statistical Analysis

Statistical analyses were performed using SPSS statistical software version 25 (IBM Corp., Armonk, NY, USA). Categorical variables were compared using the Chi-squared and Fisher's exact tests. Analysis of variance (ANOVA) was used for continuous variables. Cardiac risk factors were evaluated by univariate logistic regression.

## Results

We identified 141 patients who met the study criteria. Forty-one patients were treated with Hypo-RT and 100 patients with Conv-RT. Baseline patient characteristics are shown in Table 1. Median follow-up was 36 months (range, 13.5–90 months) in Hypo-RT group and 32 months (range, 13–89.5 months) in Conv-RT group (*p* = 0.243). The median age was 54 years (range, 38–78 years) in Hypo-RT group and 53 years (range, 29–83) in the Conv-RT group (*p* = 0.334). Laterality of the disease was similarly distributed in both treatment groups, with 51% of patients in the Hypo-RT group and 54% of patients in the Conv-RT group having left-sided disease (*p* = 0.316). The most common cardiac risk factors in both the Hypo-RT group and the Conv-RT group were BMI ≥30 (49 and 41%, respectively), age ≥55 years (46 and 38%, respectively), hypertension (46 and 33%, respectively), and smoking (34 and 23%, respectively). Patients in the Hypo-RT group had a significantly higher rate of cardiac risk factors (*p* < 0.001).

**Table 1:** Baseline characteristics.

	**Hypo-RT**	**Conv-RT**	***p***
No. patients	41	100	
Race (%)			0.053
White	26 (63)	74 (74)	
Black or African American	6 (15)	17 (17)	
Asian	9 (22)	9 (9)	
Age (y)			0.334
Median (range)	54 (38–78)	53 (29–83)	
Breast laterality (%)			0.316
Left	21(51)	54 (54)	
Right	20 (49)	46 (46)	
Histology (%)			0.786
IDC	40 (98)	95 (95)	
ILC	1 (2)	5 (5)	
Stage (%)			0.968
IA	21 (51)	17 (17)	
IIA	13 (32)	26 (26)	
IIB	4 (10)	27 (27)	
IIIA	1 (2)	15 (15)	
IIIB	2 (5)	10 (10)	
IIIC	0	5 (5)	
Estrogen receptor (%)			0.412
Positive	20 (49)	58 (58)	
Negative	21 (51)	42 (42)	
Progesterone receptor (%)			0.679
Positive	19 (48)	48 (48)	
Negative	21 (51)	50 (50)	
Unknown	1 (2)	2 (2)	
Cardiac risk factors			<0.001
BMI ≥ 30	20 (49)	41 (41)	
Age ≥ 55 years	19 (46)	38 (38)	
Hypertension	19 (46)	33 (33)	
Hyperlipidemia	10 (24)	18 (18)	
Diabetes	7 (17)	14 (14)	
CAD	1 (2)	4 (4)	
Smoking	14 (34)	23 (23)	
Family history	4 (10)	2 (2)	
Follow-up (months)			0.243
Mean	36	32	
Range	13.5–90	13–89.5	

*Hypo-RT, hypofractionated breast radiotherapy; Conv-RT, conventionally fractionated radiotherapy; IDC, invasive ductal carcinoma; ILC, invasive lobular carcinoma; BMI, body mass index; CAD, coronary artery disease*.

Treatment details are summarized in Table 2. Sixteen (39%) patients in Hypo-RT and 57 (57%) in Conv-RT group received neoadjuvant chemotherapy. The majority of patients (86% in Hypo-RT and 63% in Conv-RT) underwent breast-conserving surgery. Of the patients who received chemotherapy, a taxane-based regimen was the most common in both treatment groups. All patients received 12 months of trastuzumab. In Hypo-RT group, about 60% of patients received a dose of 42.56 Gy in 16 daily fractions. About 40% received a 3-week hypofractionated regimen of 36.63 Gy in 11 fractions followed by a 13.32 Gy boost in 4 fractions to the lumpectomy cavity on an institutional protocol. In the Conv-RT group, all patients received 5,000 cGy in 25 fractions. Over 90% of patients in both groups received a lumpectomy boost. Regional nodal irradiation was more common in the Conv-RT group (75% vs. 11%, *p* < 0.001). Mean heart dose was 101 cGy in Hypo-RT group and 163 cGy in Conv-RT group (*p* = 0.897).

**Table 2:** Treatment-related characteristics.

	**Hypo-RT**	**Conv-RT**	***p***
**Type of breast surgery**			<0.001
Breast conserving surgery	35 (86)	63 (63)	
Mastectomy with reconstruction	5 (12)	15 (15)	
Mastectomy without reconstruction	1 (2)	22 (22)	
**Systemic therapy**^**a**^			
Neoadjuvant chemotherapy	16 (39)	57 (57)	<0.001
Anthracycline based	5 (12)	20 (20)	
Taxane based (no anthracyclines)	11 (24)	37 (37)	
Trastuzumab with neoadjuvant chemotherapy	15 (37)	57 (57)	
Adjuvant chemotherapy	28 (68)	41 (41)	<0.001
Anthracycline based	2 (5)	14 (14)	
Taxane based (no anthracyclines)	26 (63)	27 (27)	
Trastuzumab with adjuvant chemotherapy	28 (68)	41 (41)	
**Endocrine therapy**	20 (49)	56 (56)	<0.001
**Radiation therapy parameters**			
Whole breast fractionation scheme (n)			
3,663 cGy in 11 fractions	16 (39)		
4,256 cGy in 16 fractions	25 (61)		
5,000 cGy in 25 fractions	0	100 (100)	
Boost, n (%)	38 (93)	91 (91)	0.550
Median dose (cGy)	1,000	1,000	
Range	1,000–1,332	800–1,600	
Radiation field design			<0.001
2-field tangents	36 (80)	25 (25)	
3–4 fields^b^	5 (11)	75 (75)	
DIBH	15 (37)	27 (27)	0.258
Heart			
Mean heart dose (cGy)	101	163	0.897
V5 (%)	1.3	4.4	0.820
V10 (%)	0.3	0.9	0.984
V20 (%)	0.1	0.3	0.967

*DIBH, deep inspiration breath hold; Vx, the percent volume receiving X Gy*.

a *Three patients in Hypo-RT group received neoadjuvant and adjuvant chemotherapy and two patients in Conv-RT group did not receive chemotherapy*.

b *Supraclavicular field with or without a posterior axillary boost*.

### Asymptomatic Changes in LVEF

**Figure 1:**
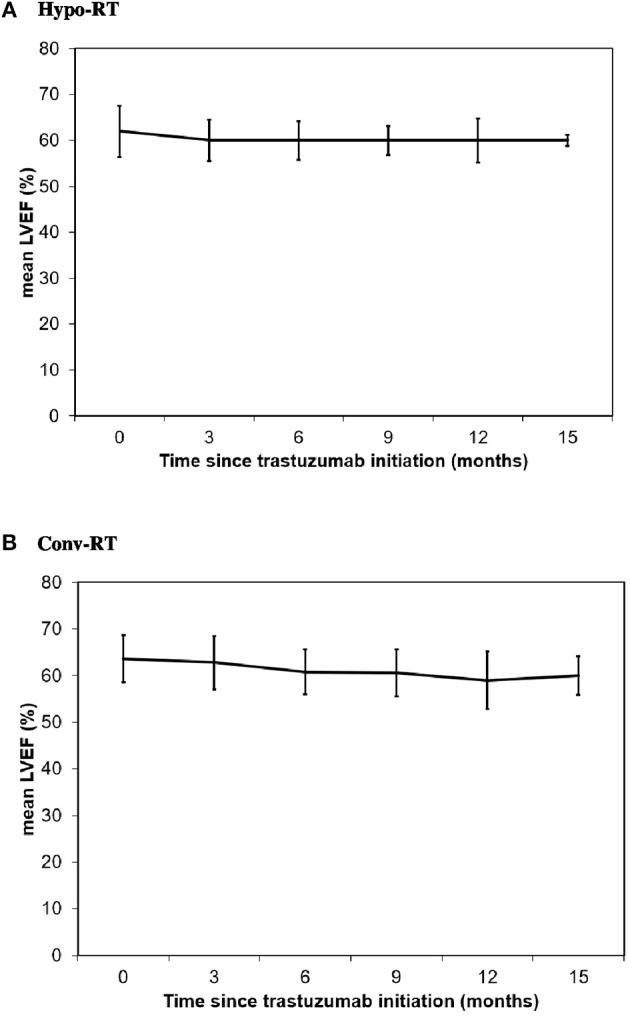
Change in LVEF from baseline in patients treated with Hypo-RT **(A)** and Conv-RT **(B)**.

Baseline median LVEF was 62% (range, 50–81%) in Hypo-RT group and 64% (range, 51–76%) in Conv-RT group (*p* = 0.893), and final median LVEF was 60% in both treatment groups (*p* = 0.998) (Table 3, Figure 1). As shown in Table 3, over 80% of patients from both groups had no decrease in LVEF from baseline or a <10% decrease in LVEF. The rate of significant asymptomatic LVEF decline (≥16% from baseline) was not significantly different between the treatment groups (7 vs. 5%, *p* = 0.203). No patients developed symptomatic CHF in either group.

**Table 3:** Change in LVEF and frequency of cardiac toxicity.

	**Hypo-RT**	**Conv-RT**	***p***
Baseline LVEF (%)			0.893
Median	62	64	
Range	50–81	51–76	
Final LVEF (%)			0.998
Median	60	60	
Range	55–72	50–75	
LVEF reduction from baseline, n (%)			0.080
No decrease	18 (44)	42 (42)	
<10%	16 (39)	40 (40)	
10–15%	4 (10)	13 (13)	
≥16%	3 (7)	5 (5)	
LVEF cardiac toxicity (%)			
Asymptomatic LVEF toxicity^*^	3 (7)	5 (5)	0.203
Symptomatic CHF	0	0	

*LVEF, left ventricular ejection fraction; CHF, congestive heart failure*.

* *Significant asymptomatic LVEF decline defined as an absolute decrease in LVEF of ≥10% to below the lower limit of normal or ≥16% from baseline value*.

Among the three patients (7%) treated with Hypo-RT who developed significant asymptomatic LVEF decline, only one had left-sided breast cancer. All three patients received a taxane-based chemotherapy regimen. Baseline LVEF was higher in all three patients compared to the median value (81, 72, and 68%). Two patients had diabetes and BMI ≥30, and one had hyperlipidemia. On univariate analysis, history of smoking (*p* = 0.307), CAD (*p* = 0.925), hypertension (*p* = 0.519), diabetes (*p* = 0.07), hyperlipidemia (*p* = 0.619), and BMI ≥30 (*p* = 0.519) had no significant effect on the development of significant asymptomatic LVEF decline. There was no significant difference in mean heart dose in patients who developed significant asymptomatic LVEF decline compared to those who did not (*p* = 0.427). Similar findings were noted for the five (5%) patients treated with Conv-RT who developed significant asymptomatic LVEF decline.

### Toxicity

Table 4 lists additional non-cardiac radiation treatment-related toxicities. Grade 2 skin toxicity was the most frequent toxicity reported in both groups (12% in Hypo-RT and 27% in Conv-RT group). All toxicities were acute and resolved during follow-up, with the exception of one patient with grade 2 lymphedema in the Hypo-RT group and two in the Conv-RT group. There were no grade 3 or 4 toxicities.

**Table 4:** Treatment-related grade 2 toxicities.

	**Hypo-RT**	**Conv-RT**
Toxicity, *n* (%)		
Skin	5 (12)	27 (27)
Fatigue	1 (2)	8 (8)
Pain	2 (5)	11 (11)
Lymphedema	1 (2)	2 (2)

## Discussion

Within a cohort of HER2-positive breast cancer patients treated with concurrent trastuzumab and Hypo-RT or Conv-RT, we note three main findings: (1) No symptomatic cardiac toxicity occurred; (2) Rates of significant asymptomatic LVEF decline in both groups were similarly low and within the range reported by randomized trials with trastuzumab; and (3) Development of asymptomatic LVEF decline was independent of multiple dosimetric cardiac parameters including mean heart dose.

Breast cancer patients have a higher rate of cardiac disease compared to age matched controls without cancer (18, 19). This increased rate of cardiac disease is due to shared risk factors for cardiac disease and breast cancer and also cancer therapy related cardiotoxicities (20–23). Traditionally, RT-associated cardiotoxicity was thought to manifest > 10 years after treatment (24–26). However, Darby et al. reported that the greatest percentage increase in the rate of major coronary artery events per Gy of heart irradiation occurred within the first 4 years after RT for breast cancer patients (11). Additionally, Marks et al. found that 6 and 12 months after RT for breast cancer, 27 and 29% of patients, respectively, had evidence of new cardiac perfusion defects (27). Furthermore, the interaction between RT-associated cardiotoxicity and trastuzumab-related cardiotoxicity is not well studied with modern adjuvant radiotherapy regimens. As hypofractionated radiotherapy is now the standard of care for the majority of women receiving whole breast irradiation, it is important to assess the safety of combining Hypo-RT with concurrent trastuzumab.

Major international trials reported the role of trastuzumab in improving survival and quality of life in HER2-positive breast cancer patients. These trials also demonstrated that cardiac events were increased with adjuvant trastuzumab (Table 5). However, the timing of trastuzumab administration was different in these trials. In the Herceptin Adjuvant (HERA) trial trastuzumab was started after both chemotherapy and RT (5), and in the Finnish Herceptin (FinHer) trial, RT was started after trastuzumab (28). In the NSABP-B31, NCCTG-9831, and Breast Cancer International Research Group 006 (BCIRG-006) trials, RT was administered with trastuzumab (6, 7).

**Table 5:** Cardiotoxicity in adjuvant trastuzumab trials.

**Trial**	**Symptomatic CHF (%)**	**Asymptomatic LVEF decline (%)**
HERA	2.1	7.4
NSABP-B31	4.1	14.2
NCCTG-9831	2.9	10.8
BCIRG-006	2	18.6
FinHer	1	3.5

*CHF, congestive heart failure; LVEF, Left ventricular ejection fraction*.

Asymptomatic LVEF decline is the most frequent form of cardiotoxicity associated with trastuzumab, with incidence rates in randomized trials ranging from 3.5 to 18.6% (Table 5). Specifically in the three large randomized trials with concurrent RT and trastuzumab, the incidence of asymptomatic LVEF decline ranged from 10.8 to 18.6%. In our study, 7% of the patients treated with Hypo-RT and 5% treated with Conv-RT experienced significant asymptomatic LVEF decline. Bonzano et al. recently compared the decrease in LVEF in HER2-positive patients treated with trastuzumab and various Hypo-RT schemes (46 Gy/20 fractions, 39 Gy/13 fractions, and 35 Gy/10 fractions) (17). Cardiotoxicities were assessed according to Common Terminology Criteria for Adverse Events v3 (CTCAE) and no difference in LVEF decline in these Hypo-RT schemes were reported. Since the assessment of LVEF decline in randomized trials is different, it is not possible to compare the result of this study with others.

During or shortly after radiotherapy, the majority of the patients experience some degree of acute RT-induced skin toxicity. The acute skin reaction is expected to be low with Hypo-RT compared to Conv-RT. During Hypo-RT, 24–27% patients experience ≥grade 2 skin toxicity (29, 30). Rates of RT-induced acute skin toxicity further decrease in those also treated with trastuzumab. Meattini et al. retrospectively reviewed 95 patients treated with Conv-RT with trastuzumab (31). Grade 2 or more acute skin toxicity was reported in 14% of the patients. In the NCCTG-9831 trial, 5.3% of the patient experienced ≥grade 3 skin toxicity (32). In our study, no grade 3 toxicity was reported and only 12% of the patients treated with Hypo-RT experienced grade 2 skin toxicity demonstrating no adverse skin toxicity associated with hypofractionated radiation.

A strength of our study is the detailed serial cardiac evaluation that was documented in the entire cohort. Although retrospective, the nearly identical baseline and serial cardiac function observed between the Hypo-RT and Conv-RT groups should be reassuring regarding the safety of administering concurrent trastuzumab and Hypo-RT in patients with breast cancer, irrespective of laterality. These data can help to reduce the use of adjuvant trastuzumab as a potential barrier to the adoption of hypofractionated radiation in patients with left sided breast cancer (12, 33). In all patients undergoing radiation, however, careful detailed simulation with deep inspiration breath hold or other techniques should be employed to limit dose to the heart to as low as is reasonably achievable. Given the low mean heart dose in our cohort, caution must be taken when extrapolating these results to facilities where such techniques are not available to decrease mean heart dose.

A limitation to our study is of course its retrospective design and inherent confounding factors that cannot be totally accounted for in a non-randomized study. In addition, the small sample size and limited follow-up do not adequately address potential long-term cardiotoxicity. However, the detailed serial cardiac function evaluations were consistent with the plethora of available literature on cardiac function changes in patients undergoing chemotherapy for breast cancer and there was no evidence that hypofractionation or conventional fractionation significantly compromised or impacted LVEF.

In conclusion, the rate of asymptomatic LVEF decline in patients receiving concurrent trastuzumab and Hypo-RT was similar to those treated with Conv-RT and was independent of mean heart dose. Longer follow-up is warranted to assess late cardiotoxicity.

## Data Availability Statement

The datasets generated for this study are available on request to the corresponding author.

## Ethics Statement

This study was approved by Ethics Committee of Rutgers Cancer Institute of New Jersey and in accordance with the Declaration of Helsinki.

## Author Contributions

All authors listed have made a substantial, direct and intellectual contribution to the work, and approved it for publication.

### Conflict of Interest

The authors declare that the research was conducted in the absence of any commercial or financial relationships that could be construed as a potential conflict of interest.
